# ChIA-PET tool for comprehensive chromatin interaction analysis with paired-end tag sequencing

**DOI:** 10.1186/gb-2010-11-2-r22

**Published:** 2010-02-25

**Authors:** Guoliang Li, Melissa J Fullwood, Han Xu, Fabianus Hendriyan Mulawadi, Stoyan Velkov, Vinsensius Vega, Pramila Nuwantha Ariyaratne, Yusoff Bin Mohamed, Hong-Sain Ooi, Chandana Tennakoon, Chia-Lin Wei, Yijun Ruan, Wing-Kin Sung

**Affiliations:** 1 Computational and Mathematical Biology, Genome Institute of Singapore, 60 Biopolis Street, Singapore, 138672, Republic of Singapore; 2 Genomic Technologies, Genome Institute of Singapore, 60 Biopolis Street, Singapore, 138672, Republic of Singapore; 3 Department of Computer Science, School of Computing, National University of Singapore, 13 Computing Drive, Singapore, 117417, Republic of Singapore; 4 Department of Biochemistry, Yong Loo Lin School of Medicine, National University of Singapore, 8 Medical Drive, Singapore, 117597, Republic of Singapore

## Abstract

Chromatin interaction analysis with paired-end tag sequencing (ChIA-PET) is a new technology to study genome-wide long-range chromatin interactions bound by protein factors. Here we present ChIA-PET Tool, a software package for automatic processing of ChIA-PET sequence data, including linker filtering, mapping tags to reference genomes, identifying protein binding sites and chromatin interactions, and displaying the results on a graphical genome browser. ChIA-PET Tool is fast, accurate, comprehensive, user-friendly, and open source (available at http://chiapet.gis.a-star.edu.sg).

## Abstract 2

ChIA-PET Tool can be used to process long-range chromatin interaction data. Results are visualized on a user-friendly genome browser.

## Rationale

Transcription factors and their three-dimensional interactions are crucial to gene regulation [1,2]. Many distal transcription factor binding sites have been identified by genome-wide chromatin experiments, such as chromatin immunoprecipitation (ChIP)-chip [3], ChIP-paired-end tag (PET) [4], and ChIP-Seq [5], but it is not clear which of these distal transcription factor binding sites are real and functional in gene regulation, and which are non-functional 'parking spots'. Three-dimensional chromatin interactions have been shown to bring distal transcription factor binding sites into close spatial proximity to gene promoters [6], but global analysis of three-dimensional chromatin interactions has been limited by the lack of techniques for high-resolution and whole-genome analysis.

**Figure 1:**
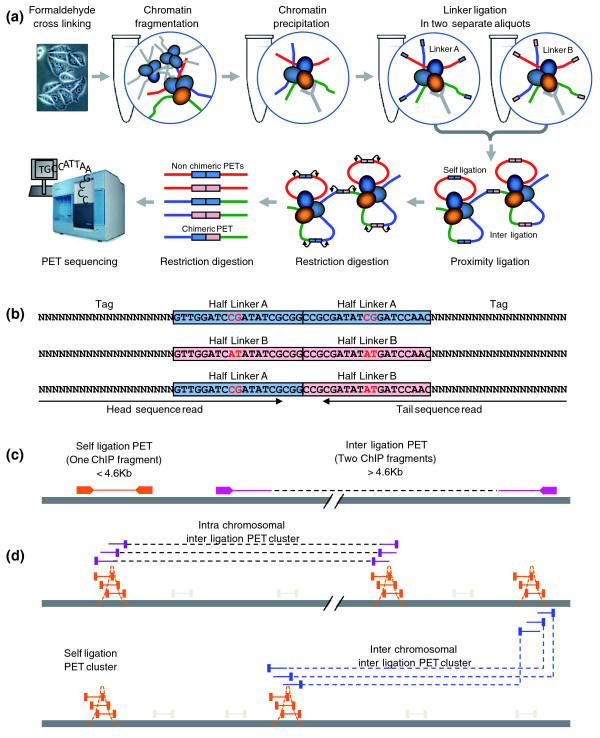
**Schematic of ChIA-PET analysis**. **(a) **The ChIA-PET experimental protocol, which includes chromatin preparation, ChIP, linker ligation, proximity ligation, *Mme*I restriction digestion, and DNA sequencing. **(b) **The ChIA-PET constructs prepared for sequencing analysis. Each PET construct involves a pair of tags (20 bp each) and a linker (38 bp) between the tag pairs. This full-length linker is derived from ligation of two half-linkers, A or B, each with a unique barcode nucleotide (CG for half-linker A and AT for half-linker B). The barcode nucleotides are highlighted as red letters. Linkers with AB barcodes are considered to be non-specific chimeric proximity ligation products. **(c) **Mapping tags of PET sequences to reference genome. The categories of 'self-ligation PETs' and 'inter-ligation PETs' were assigned. **(d) **Clustering of overlapping PET sequences in the same genomic regions to identify enriched protein binding sites by overlapping 'self-ligation PETs' and long-range chromatin interactions by overlapping 'inter-ligation PETs'.

Recently, we developed a global, *de novo*, high-throughput method, Chromatin interaction analysis with paired-end tag sequencing (ChIA-PET), for characterizing the three-dimensional structures of long-range chromatin interactions in the nucleus [7-9], which makes it possible to identify transcriptional binding sites involved in long-range interactions at a genome-wide level. The key features in ChIA-PET analysis (Figure 1a) are that the cross-linked chromatin interaction nodes bound by protein factors are enriched by ChIP, and remote DNA elements tethered together in close spatial distance in these chromatin interaction nodes are connected through proximity ligation with oligonucleotide DNA linkers. We designed linker sequences that not only contain *Mme*I restriction sites for PET extraction, but also include specific nucleotide barcodes to assess the noise level in ChIA-PET data from random ligation. Upon *Mme*I digestion, the resulting PET construct contains a 20 bp head tag, a 38 bp linker sequence, and a 20 bp tail tag, which is the template for next generation paired-end sequencing, for example, Illumina paired-end sequencing from the two ends in opposite directions (Figure 1b). Each of the paired sequencing reads uncovers the 20 nucleotide tag sequence and the 16 nucleotide sequence from the attached linker sequence including the nucleotide barcodes. When PETs are mapped to the corresponding reference genome sequences, the genomic distance between the two mapped tags will reveal whether a PET is derived from a self-ligation product of a single DNA fragment (short genomic distance) or an inter-ligation product of two DNA fragments (long genomic distance, or inter-chromosomal) (Figure 1c). The overlapping ChIP fragments inferred by PET sequences will reveal true binding sites and long-range chromatin interactions bound by such protein factors, whereas the singletons mostly reflect the random background noise (Figure 1d).

The ChIA-PET approach is very efficient in generating large volumes of PET sequence data for long-range chromatin interactions with different protein factors in complex genomes. Since the detection of long-range chromatin interactions involves high levels of background noise due to the complexity of chromatin structures in nuclear space and the nature of proximity ligation [7,8], a meaningful analysis requires a comprehensive, efficient pipeline. The immense challenges in the setup of an efficient pipeline to process the huge body of ChIA-PET sequence data include: how to accurately filter the linker sequences from the raw reads; how to accurately and efficiently map the tag sequences to reference genomes; how to evaluate the noise level in the data; how to identify *bona fide *binding sites and chromatin interactions; how to organize the datasets; and how to effectively visualize the long-range chromatin interactions identified by ChIA-PET analysis. Many of the bioinformatics challenges faced in the ChIA-PET analysis are unprecedented.

In developing the ChIA-PET data analysis algorithms, we assembled a package of sophisticated bioinformatics solutions called 'ChIA-PET Tool' for processing, analyzing, visualizing, and managing ChIA-PET data quickly, accurately, and automatically. In this report, we describe the design and implementation of ChIA-PET Tool, and demonstrate its efficiency and effectiveness through processing and analyzing an estrogen receptor α (ERα) ChIA-PET library dataset from the MCF-7 cell-line.

## The architecture design of ChIA-PET Tool

**Figure 2:**
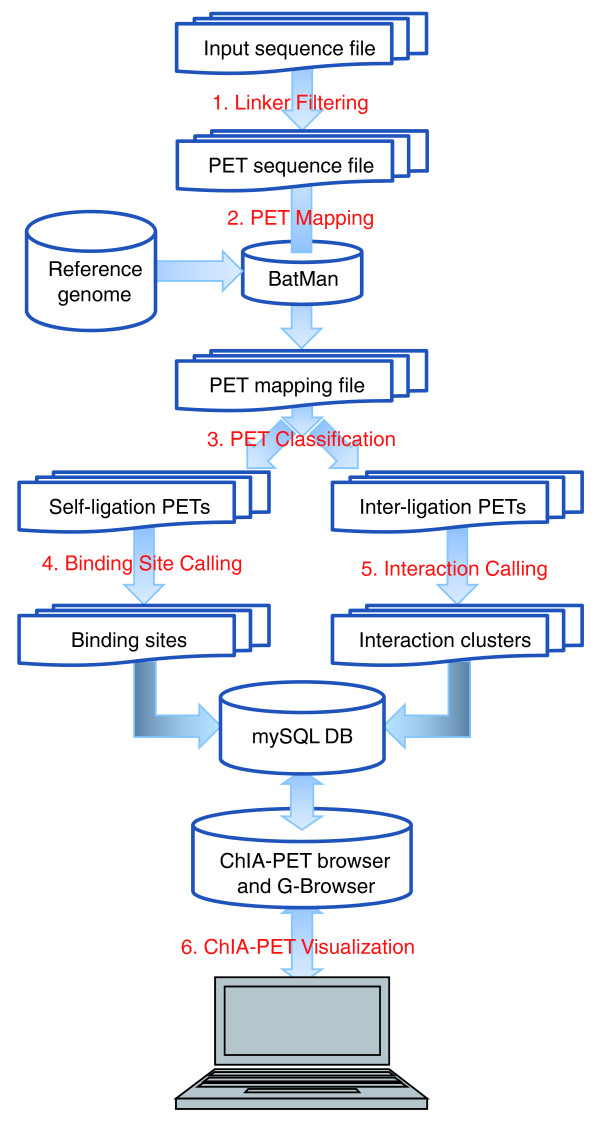
**Architecture of ChIA-PET Tool**. The six main modules in ChIA-PET Tool (labeled) connect the input sequence data, the intermediate data, and the final results. The details of the processing are referred to in the main text.

The architecture of ChIA-PET Tool includes six modules: Linker filtering, PET mapping, PET classification, Binding site calling, Chromatin interaction calling, and ChIA-PET visualization (Figure 2). First, in the Linker filtering module, the linkers from the input sequence reads are determined, and the PETs are sorted by the presence of readable linker barcodes. The PETs without readable linker barcodes are assigned as 'ambiguous PETs', whereas the PETs with readable barcodes are further assigned into chimeric PETs if they have heterogeneous linker barcode compositions (AB linker) or non-chimeric PETs if they have homogenous barcode compositions (AA or BB) (Figure 1b). Next, in the PET mapping module, the PET sequences are mapped to the corresponding reference genome. The mapped PETs are then classified based on the genomic spans of the two tag mapping locations into 'self-ligation PETs' (short genomic spans) and 'inter-ligation PETs' (long genomic spans or inter-chromosomal). The self-ligation PETs are used for calling putative binding sites, and the inter-ligation PETs are used for chromatin interaction analysis. The processed results are uploaded to a mySQL database for organization and visualization in ChIA-PET browser and the generic genome browser (G-browser) [10].

**Figure 3:**
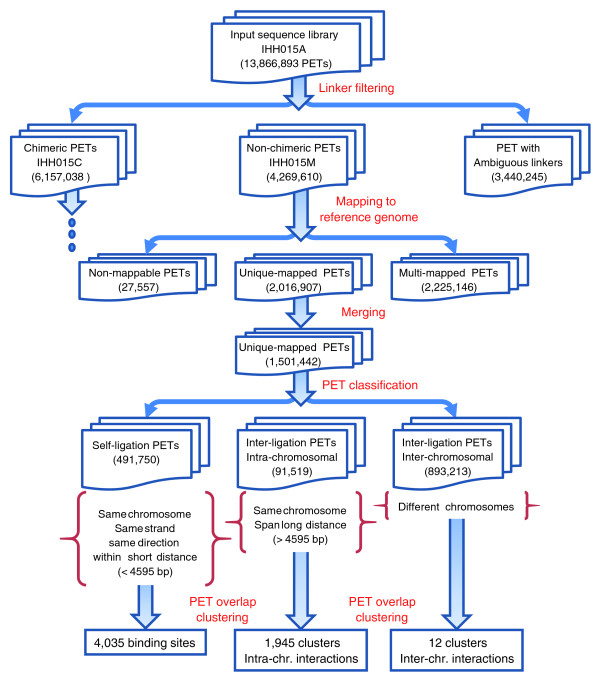
**ChIA-PET data analysis flow of library IHH015A**. A library, IHH015A, is used to demonstrate the ChIA-PET Tool data analysis flow, and the non-chimeric data IHH015M from IHH015A is used to show the analysis results.

To demonstrate the analysis procedure of ChIA-PET Tool, we used a real ChIA-PET library, IHH015A, for illustration. IHH015A is a part of the datasets of an ERα ChIA-PET study reported previously [9]. This ChIA-PET library consists of 13,866,893 PETs generated by Illumina GAII paired-end sequencing, and was separated into chimeric PETs (IHH015C) and non-chimeric PETs (IHH015M) through the linker filtering procedure described below. The analysis results are summarized in Table 1 and the remaining data analysis flow is mainly illustrated with the non-chimeric library IHH015M (Figure 3).

**Table 1:** Statistics of ChIA-PET data analysis

	IHH015A^a^	IHH015M^b^	IHH015C^c^
Total PETs	13,866,893	4,269,610 (30.8% of IHH015A)	6,157,038 (44.4% of IHH015A)
Unique PETs with unique mapping	3,826,699	1,501,442	1,710,335
Self-ligation PETs	611,622	491,750	12,155
Different orientation PETs	31,543	24,960	548
Intra-chromosomal inter-ligation PETs (excludes different orientation PETs)	223,170	91,519	94,743
Inter-chromosomal inter-ligation PETs	2,960,364	893,213	1,602,889
Binding sites (FDR <0.01, filtered^d^)	5223	4,035	24
Intra-chromosomal interactions (FDR <0.05; PET cluster size ≥ 3; filtered^d^)	2221	1,945	12
Inter-chromosomal interactions (FDR <0.05; PET cluster size ≥ 3; filtered^d^)	18	12	0

^a^The original ChIA-PET dataset without linker filtering. ^b^The dataset containing non-chimeric linker compositions AA and BB. ^c^The dataset containing chimeric linker composition AB, considered as non-specific proximity ligation noise. ^d^The binding sites and interactions after filtering those from chromosome Y, the mitochondria, and satellite repeat regions, and interactions from genome structural variations in the MCF-7 cell-line. FDR, false discovery rate.

### Linker filtering

A ChIA-PET input sequence has a pair of reads from the two opposite directions of the same ChIA-PET template. Both reads are 36 nucleotides long, and contain 20-nucleotide tag information and 16-nucleotide linker information (Figure 1b). To determine the linker type, we aligned the linker part of the reads (the last 16 nucleotide sequence of the 36 nucleotides) to the half-linker nucleotide sequence A (or B). If both the paired reads have good alignment with half-linker A (or B) and the specific nucleotide positions 9 and 10 (the nucleotide barcode) are CG (or AT), we classify the PET as having a homogenous full linker composition with AA (or BB). If one read in a PET has a good alignment with the half-linker A while the other one has a good alignment with the half-linker B, we classify this PET as having a heterogeneous full linker composition with AB (or BA). If there is no good alignment with any of the half-linkers (could also indicate low sequence quality), the PETs are classified as ambiguous PETs and will be discarded from further analyses. A PET sequence with a full linker AB indicates that this PET is derived from a ligation product formed between two different ChIP complexes from the two separate half-linker aliquots (Figure 1a). Therefore, the corresponding PETs with linker composition AB are most likely derived from random and non-specific ligations between two different ChIP complexes. Hence, we classified the PETs with linker AB as the chimeric PET dataset, and the PETs with linkers AA and BB as non-chimeric PET dataset (note that the PETs with linkers AA and BB may still have certain chimeric PETs). After the linker alignment, the linker parts in the short sequence reads will be trimmed and the tag sequences will proceed for further analyses.

With linker filtering, ChIA-PET library IHH015A data were divided into two pools: IHH015M (mix of PETs with AA and BB linkers) and IHH015C (chimeric PETs with AB linkers). The IHH015M dataset has 4,269,610 PETs (30.8% of total input PET sequences) and IHH015C has 6,157,038 PETs (44.4%). The remaining PETs were classified as PETs with ambiguous linkers.

### PET mapping to reference genome

After linker trimming, the tags are mapped to the corresponding reference genome using the Batman package (C Tennakoon *et al.*, manuscript submitted) with at most one mismatch. Batman is a Burrows-Wheeler-transform-based method [11] that maps short sequences to a genome with very high speed. For each tag, Batman first considers the exact matches to the reference genome. If a single exact match is obtained, that location is taken as the mapping location of the tag, and the tag is classified as 'unique mapping'. If multiple exact matches are obtained, the tag is classified as 'multiple mappings'. If no exact match is obtained for the tag, a mapping is done with one mismatch allowed, and the result is similarly labeled as 'unique mapping' or 'multiple mappings'. If there is still no mapping for a tag with one mismatch, the tag is finally classified as 'non-mappable'.

After mapping the tags to the human reference genome (hg18) with Batman, only those PETs from IHH015M and IHH015C with both tags uniquely mapped to the reference genome were considered for further analyses. The remaining PETs with tags multiply mapped or unmapped to the reference genome were filtered out. We obtained 2,016,907 PETs in IHH015M and 2,707,860 PETs in IHH015C with unique mappings. To further avoid miscalling of clonal amplifications by PCR involved in sample preparation as enrichment of ChIP fragments, we merged all similarly mapped PETs (within ± 1 bp) into one unique PET. In this way, we reduced false positive calls resulting from the same PCR clonal amplification. Finally, we obtained 1,501,442 unique PETs with unique mapping from IHH015M and 1,710,335 unique PETs with unique mapping from IHH015C.

### PET classification

The ChIA-PET sequences can be classified into two categories: self-ligation and inter-ligation PETs (Figure 1c). 'Self-ligation PETs' are obtained from self-circularization ligation of the same chromatin fragments, and result in ChIA-PET sequences with both tags mapped within a short genomic distance of each other on the same chromosome in a head-to-tail orientation. 'Inter-ligation PETs' are derived from inter-ligation between two different DNA fragments, and can be partitioned into three different sub-categories: 'inter-chromosomal inter-ligation PETs', 'intra-chromosomal inter-ligation PETs', and 'different-orientation ligation PETs'. 'Inter-chromosomal inter-ligation PETs' are PETs with two tags mapped to two different chromosomes. 'Intra-chromosomal inter-ligation PETs' are PETs with both tags mapped to the same chromosome with a long genomic span, since PETs with long genomic span cannot arise from individual short chromatin fragments. 'Different-orientation ligation PETs' are PETs with both tags mapped to the same chromosome within a short genomic span, but with the wrong orientation or on different strands.

**Figure 4:**
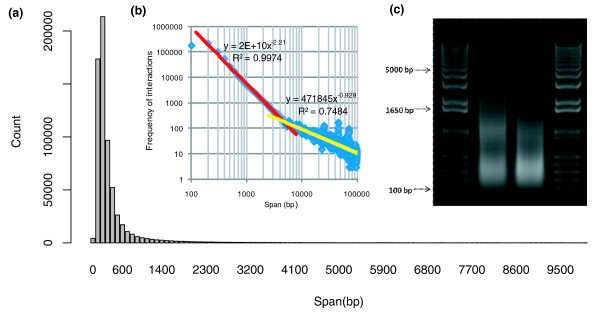
**Span distribution of intra-chromosomal PETs and the cutoff between self-ligation PETs and inter-ligation PETs**. **(a) **The distribution of the intra-chromosomal PET genomic spans. **(b) **The log-log plot of interaction frequencies against PET span, and the cutoff between self-ligation PETs and inter-ligation PETs. **(c) **Agarose gel of chromatin DNA fragments prepared for ChIA-PET analysis. Selected DNA sizes are marked.

To determine the span cutoff between self-ligation and intra-chromosomal inter-ligation PETs, we plotted the genomic spans of the PETs mapped on the same chromosomes of the IHH015A dataset. The histogram shows that the vast majority of these PETs do not have genomic span over 2 kb (Figure 4a). Similarly, using log-log plot analysis of the same data (logarithm frequency against the logarithm span), we observed a mixture model with two straight distribution lines (Figure 4b), clearly representing two distinctive PET populations. The chromatin DNA was randomly chopped into short fragments (represented by self-ligation PETs) by sonication, which can be modeled by a 'stick-breaking process' [12] for breaking long chromatin fibers. Our analysis and simulation suggest that the size distribution of the chromatin DNA fragments represented by self-ligation PETs follows a power-law distribution, which is a straight line in a log-log plot and corresponds to the left line in Figure 4b. By contrast, the right line in Figure 4b represents a PET population clearly different from the self-ligation PETs, which follows another power-law distribution and is an approximation for the intra-chromosomal chromatin interaction model as suggested by Dekker *et al. *[13]. Therefore, the span cutoff between self-ligations and intra-chromosomal inter-ligations can be determined by the intersection of the two lines in the log-log plot. In our analysis for the IHH015M library data, the span cutoff called by our method is 4,595 bp. This estimation for DNA fragment size is consistent with agarose gel electrophoresis of the chromatin DNA sonication profile (Figure 4c) in the ChIA-PET protocol. The agarose gel result in Figure 4c clearly shows that most DNA fragments are below the 1,650 bp mark and it is hard to see any DNA smear above 5,000 bp.

Accordingly, the PET datasets in IHH015M and IHH015C were classified into different categories. In IHH015M, 491,750 PETs were classified as self-ligation PETs. By contrast, IHH015C only yielded 12,155 self-ligation PETs. IHH015M contains 91,519 intra-chromosomal inter-ligation PETs and 893,213 inter-chromosomal inter-ligation PETs. IHH015C contains 94,743 intra-chromosomal inter-ligation PETs and 1,602,889 inter-chromosomal inter-ligation PETs. We note that the number of self-ligation PETs in IHH015M (with homogenous linker AA and BB; non-chimeric) is more than 40 times (491,750/12,155 = 40.5) that of the self-ligation PETs in IHH015C (with heterogeneous linker AB; chimeric). This is expected because self-ligations of the same DNA fragments are supposed to have the same linker types based on the experimental design (Figure 1a, b).

To check whether the inter-ligation PETs in IHH015M and IHH015C arise due to random ligation, the ratio of the intra-chromosomal inter-ligation PETs and the inter-chromosomal inter-ligation PETs was analyzed with a random model. From the PET datasets IHH015M and IHH015C, the numbers of DNA fragments from each chromosome were counted. Assuming that the ligation of the fragments occurs in a random manner and one fragment has an equal chance of ligating to any other fragments, the expected rate of finding an intra-chromosomal inter-ligation PET in a specific chromosome is proportional to the square of the number of DNA fragments in this chromosome. The total rate of intra-chromosomal inter-ligation PETs is proportional to the sum of the square of the numbers of DNA fragments over all individual chromosomes. Therefore, based on a random model, the expected rate of intra-chromosomal inter-ligation PETs is 0.0552 for IHH015C and 0.0546 for IHH015M. By contrast, the observed rate was 0.0558 for IHH015C and 0.0929 for IHH015M. The fold change between the observed rate and the expected rate for IHH015C (0.0558/0.0552 = 1.01; *P*-value 5.2E-4 from binomial test) was insignificant, validating the notion that the chimeric inter-ligation PETs are derived from random ligation. By contrast, the difference between the observed rate and the expected rate for IHH015M (0.0929/0.0546 = 1.70; *P*-value < 2.97E-323) was very significant, suggesting that the non-chimeric inter-ligation PETs are not random and probably enriched for specific chromatin interactions.

**Figure 5:**
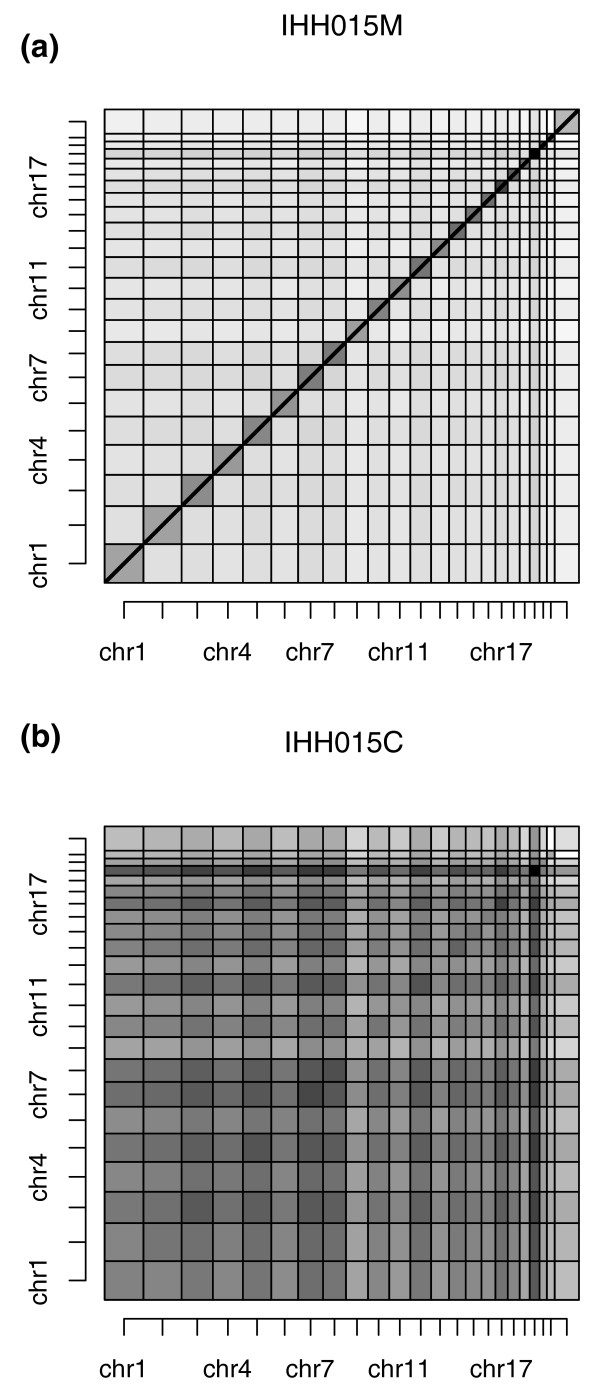
**Genome-wide ChIA-PET interaction density plots from the IHH015M and IHH015C PET datasets The square density plot in each graph is normalized to [0, 1]**. Darker squares indicate higher interaction densities.

To visually illustrate the differences between the ChIA-PET libraries IHH015M and IHH015C, we represented every PET in these two datasets as a point (x, y) on a two-dimensional map where x and y axes are the genome loci of the two tags in a PET. Figure 5. shows the density map of all the PETs in the two ChIA-PET libraries, by normalizing the maximum density to 1. The darker the rectangle, the higher is the PET density between two corresponding chromosomes. For the chimeric PET dataset (IHH015C), there was no particular distribution pattern of PET density. By contrast, for the non-chimeric PET dataset (IHH015M), the PET density of the intra-chromosomal inter-ligations was much higher than that of the inter-chromosomal inter-ligations, suggesting that most potential chromatin interactions detected by ChIA-PET are intra-chromosomal, not inter-chromosomal.

In summary, our analyses showed that non-chimeric PET dataset IHH015M is significantly different from the random model and the data can be used for further analyses.

### Binding site analysis

Binding sites can be identified by looking for clusters of overlapping self-ligation PETs. As background noise is random, noisy PETs should distribute randomly throughout the genome. Only ChIP-enriched fragments would form clusters of overlapping self-ligation PETs, and are considered putative binding sites (Figure 1d). The probability that a cluster contains *k *self-ligation PETs by random chance can be calculated by a Monte Carlo simulation, allowing false discovery rates to be assigned to different clusters. The clusters with false discovery rates below a particular threshold, such as 0.01, are putative binding sites. A similar method was previously applied in ChIP-PET data analysis [4]. In considering the ChIP enrichment score of a binding site, the number of ChIP fragments at a binding site is counted. This is similar to the existing ChIP enrichment calculation protocols in ChIP-Seq data [5].

After predicting binding sites, a post-processing step can be applied to remove suspicious binding sites, which can arise from different sources. For example, a library from female cells, such as MCF-7, is not expected to have any binding sites in chromosome Y. Binding sites in satellite repeat regions are also likely to be attributable to non-specific mapping and should be dropped. Further, certain binding sites in cancer genomes may be the results of genome amplifications [8,14], and should be flagged, such that caution can be exercised in using them.

In our analysis, after calling putative binding sites and removing binding sites likely to be due to non-specific mapping, 4,035 binding sites were called from the IHH015M dataset at a false discovery rate <0.01, which covered 47,735 (9.7%) of the 491,750 self-ligation PETs in IHH015M. By contrast, only 24 binding sites were called from the chimeric PET dataset IHH015C at the same false discovery rate, which covered only 130 (1.1%) of 12,155 self-ligation PETs in IHH015C. This indicates that most of the binding sites of IHH015M are *bona fide*. Indeed, many of the ERα binding sites identified in IHH015 library were validated using ChIP-qPCR [9].

### Chromatin interaction analysis

Chromatin interaction identification is done in two steps: first, define the ChIP enriched interaction anchor regions from inter-ligation PETs and then quantify the interaction frequency by counting the number of inter-ligation PETs between the two connected anchor regions. The identification of ChIP-enriched interaction anchors from inter-ligation PETs is performed by finding peaks and valleys from the overlapped tags from the inter-ligation PETs, in a similar manner employed by ChIP-Seq [15]. The tag length of each inter-ligation PET is extended in a 5' to 3' manner by the 'tag extension length' to represent a ChIP DNA fragment. The 'tag extension length' is equivalent to the most frequently detected span of the self-ligation PETs, which is around 200 bp for the IHH015A library. Most of the interaction anchor regions identified from inter-ligation PETs should also be overlapped with the protein binding sites identified from self-ligation PETs. After defining the enriched anchor regions from inter-ligation PETs, the number of overlapping inter-ligation PETs between any two anchors is counted to reflect the relative interaction frequency. As each interaction involves two anchors and one loop, it is called a 'duplex interaction'. Similar to the binding site analysis, a real interaction is expected to involve multiple overlapping inter-ligation PETs connecting two anchors.

To determine if an interaction PET cluster between two anchors is a real chromatin interaction and not by random chance, a simple method is to count the number of inter-ligation PETs in the interaction cluster. If the cluster has a higher PET count, it has a higher probability to be a real chromatin interaction. This model, however, does not take into account the fact that, when the ChIP enrichments of two anchors are high, there is also a higher probability to form more inter-ligation PETs by random chance between these two anchors. To address such concerns, we formulated a statistical analysis framework to account for the random formation of any inter-ligation PETs between two anchors. The null hypothesis assumes that, in the ChIP-enriched chromatin fragment population, each chromatin fragment has an equal chance to ligate to any other fragments in a random manner, and the interactions between different anchors are independent of each other. Under this random model, the number of inter-ligation PETs that link two anchors follows a hyper-geometric distribution. The formula is provided in Equation 1:(1)

The expected interaction frequencies between any two genomic loci and the false discovery rate for each interaction were calculated. Hence, both inter-ligation PET frequency and ChIP enrichment of the anchors are taken into account by this analysis.

**Figure 6:**
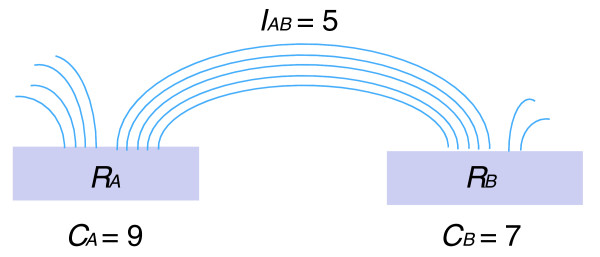
**An illustration of the statistical model for probability analysis of ChIA-PET interactions**. *R*_*A *_and *R*_*B *_represent two DNA regions ('anchors') with *c*_*A *_and *c*_*B *_PETs (here 9 and 7, respectively). *I*_*A*, *B *_is the number of inter-ligation PETs between *R*_*A *_and *R*_*B *_and here *I*_*A*, *B *_is equal to 5.

Equation 1 considers a library with *N *inter-ligation PETs. *R*_*A *_and *R*_*B *_represent two anchor regions with *c*_*A *_and *c*_*B *_PETs, respectively, where *c*_*A*_, *c*_*B *_<<*N*. Equation 1 shows that, when *c*_*B *_ends are randomly chosen from 2*N *ends as ends in region *R*_*B*_, what is the probability of choosing *I*_*A*, *B *_ends from *c*_*A *_ends of region *R*_*A *_to form *I*_*A*, *B *_interactions between region *R*_*A *_and region *R*_*B*_. By this, we are able to compute a *P*-value to test if *I*_*A*, *B*_, the number of inter-ligation PETs between *R*_*A *_and *R*_*B*_, is over-represented. Given a cut-off threshold, T, of hypergeometric *P*-value, we are able to calculate the false discovery rate, which is the fraction of the clusters with *P*-value below T under the empirical random model generated by randomly permuting the ends of PETs. As *c*_*A *_and *c*_*B *_reflect the ChIP enrichment of two DNA anchors, any ChIP enrichment bias is accounted for by this model. An illustration of the random model is shown in Figure 6.

From the predicted interactions with three or more inter-ligation PETs between anchors and false discovery rate < 0.05, we filtered the interactions that could be a result of mis-mapping or noise, as we did with the binding sites. We filtered out any interactions wherein the anchors were present in chromosome Y or the mitochondria, and satellite repeat regions. We also filtered out a specific kind of noise in the interaction clusters from genome structural variations in cancer cell-lines. The cancer cell-lines like MCF-7 have intensive genome rearrangements: genome insertion, deletion, inversion, and translocation, which can be identified with DNA-PET data [8,14]. Self-ligation PETs around the breakpoints of genome rearrangement from cancer cell-lines will be mapped as inter-ligation PETs in the reference genome, and interaction clusters related to such genome rearrangements should be removed.

**Figure 7:**
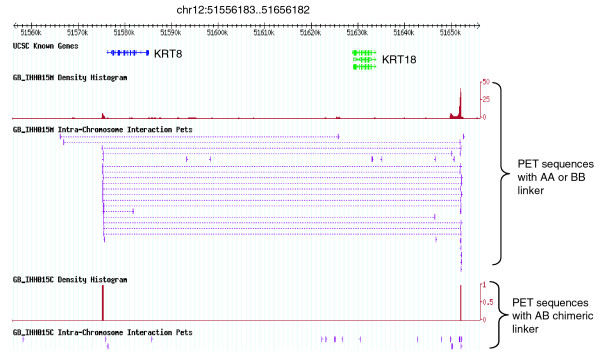
**An example of ChIA-PET mapping display in G-browser**. A screenshot of the ChIA-PET G-browser is shown in human chromosome 12 at the keratin gene family region. The tracks are (from top to bottom): UCSC known genes track, which shows the KRT8 and KRT18 genes in blue and green; density histogram of IHH015M self-ligation PETs, which consists of AA and BB PET sequences, wherein peaks indicate enriched ERα-binding sites; intra-chromosomal inter-ligation PETs of IHH015M, wherein multiple overlapping inter-ligation PETs indicate interactions and singleton inter-ligation PETs indicate noise; density histogram of IHH015C self-ligation PETs that consists of only chimeric (AB) PET sequences; intra-chromosomal inter-ligation PET tracks of IHH015C. From the graph, we know that IHH015M contains binding sites and interactions, and IHH015C does not, although both libraries have similar sequencing depth.

Using the above analysis procedure and a threshold of 3 or more for the number of overlapping inter-ligation PETs, we identified 1,945 putative intra-chromosomal interactions and 12 putative inter-chromosomal interactions in the IHH015M dataset. Our validations, including 3C [13], ChIP-3C [16], and 4C [17], as well as fluorescent *in situ *hybridization (FISH) and small interfering RNA experiments, suggest that the majority of the intra-chromosomal chromatin interactions identified in this analysis are *bona fide *loci bound by ERα as reported in Fullwood *et al. *[9]. By contrast, the chimeric inter-ligation PETs in IHH015C yielded only 12 intra-chromosomal and zero inter-chromosomal inter-ligation PET clusters. Detailed manual curation verified that none of them constitute real interactions. Our comparison of a non-chimeric ChIA-PET dataset (IHH015M) with a similarly sized chimeric ChIA-PET dataset also indicates that the non-chimeric dataset generates statistically significant binding sites and interactions, whereas the chimeric dataset does not. This means that chimerism is not an issue in obtaining *bona fide *binding sites and chromatin interactions in the ChIA-PET library. As an example, abundant non-chimeric inter-ligation PETs in the IHH015M dataset identified the ERα-bound chromatin interaction event at the KRT8/18 locus in the human genome (Figure 7.), but no chimeric PETs in the IHH015C dataset were found at the KRT8/18 locus. This interaction at the KRT8/18 locus has been validated by 4C experiments [9].

### ChIA-PET data visualization

**Figure 8:**
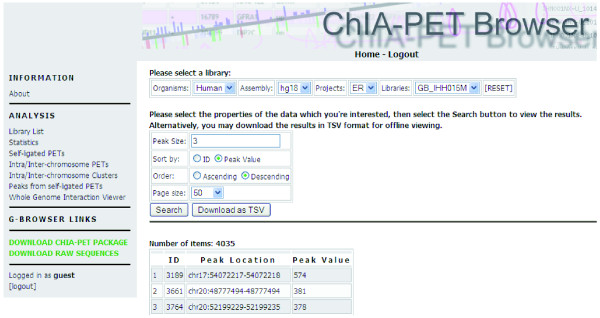
**Screen shot of binding site table view in ChIA-PET browser**.

**Figure 9:**
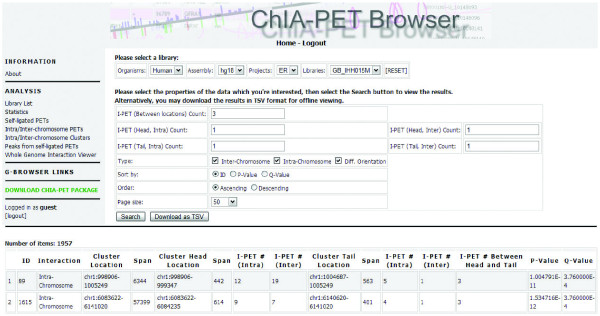
**Screen shot of interaction cluster table view in ChIA-PET browser**.

**Figure 10:**
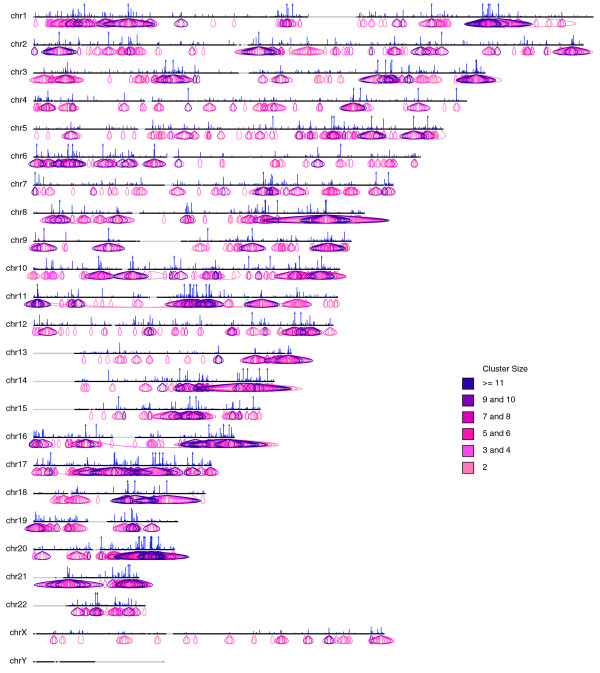
**Screen shot of the whole genome interaction view in ChIA-PET browser**.

All ChIA-PET data, including the called binding sites and chromatin interaction clusters, are uploaded to a mySQL database. A centralized ChIA-PET browser is built to organize data reporting and visualization. The ChIA-PET browser consists of two components: a tabular browser and a graphic genome browser. The tabular browser provides an organization of all ChIA-PET experimental datasets (libraries) from a variety of projects. It reports the unique PETs with unique mapping, the binding sites and the interaction clusters in tabular forms, and the users can download the data for further analysis. Example screenshots of binding sites, interaction clusters and the whole genome interactions from IHH015M are provided in Figures 8, 9 and 10. The graphical genome browser (G-browser) is created by adopting the 'generic genome browser system' [10], which allows users to view and manually curate the data. A screenshot of the G-browser is shown in Figure 7 More details of the browsers can be found from the ChIA-PET website [18] (username, guest; password, guest).

### Implementation and performance

ChIA-PET Tool is implemented with various software written in C, Java and scripting languages (PERL and Python), and has been tested with the Linux operating system. The components in the pipeline are linked up together to behave as a singular processing pipeline. The pipeline requires hardware support; for example, 32 Gbytes RAM and 1,024 Gigabytes hard disk are recommended on a duo-core server. mySQL (5.0.67 or higher) is used as the database engine. Other required software packages are Apache, Perl and Bioperl modules, and PHP (refer to the ChIA-PET Tool installation guide on the ChIA-PET website [18] for details).

We have tested the ChIA-PET Tool pipeline with two hardware configurations: one with 32 Gbytes RAM and 8 CPUs, and another with 64 Gbytes RAM and 16 CPUs. The input to the pipeline is the original paired-end sequence data from the Illumina sequencing output file. A ChIA-PET library from a single lane requires approximately 1 Gigabyte space for storage, which includes intermediate files generated throughout the processing. On average, it takes 1 hour for ChIA-PET Tool to process a single lane ChIA-PET dataset and 1 hour to upload the GFF file to the G-browser database (the upload time is subject to existing database size and server load).

## Conclusions

We developed a comprehensive computational package, ChIA-PET Tool, to accommodate large amounts of ChIA-PET data, process the linkers, map the short tags to the reference genomes, classify the PETs into different categories, and identify statistically significant binding sites and chromatin interactions. We demonstrate the effectiveness of ChIA-PET Tool by analyzing a ChIA-PET library IHH015A with statistical results, and show that ChIA-PET Tool is a convenient, user-friendly, accurate bioinformatics solution, and an integral component of the ChIA-PET process for chromatin interaction analysis. Although we have only reported the use of ChIA-PET Tool in ERα ChIA-PET analysis, it is obvious that this tool can be applied to different ChIA-PET libraries bound by different transcription factors in different genomes for chromatin interaction analysis.

## Availability

ChIA-PET Tool is open-source and free for non-commercial use. The complete package of ChIA-PET Tool is downloadable from the ChIA-PET website [18], together with the ChIA-PET Tool file format, the ChIA-PET Tool installation guide, the ChIA-PET Tool user manual, and the ChIA-PET browser user manual. The raw sequences and the processed data are also available from ChIA-PET website [18] (username, guest; password, guest). More related data for ChIA-PET analysis are accessible from NCBI's Gene Expression Omnibus with accession number [GEO:GSE18046].

## Abbreviations

bp: base pair; ChIP: chromatin immunoprecipitation; ChIA-PET: chromatin interaction analysis with paired-end tag sequencing; ERα: estrogen receptor α; G-browser: generic genome browser; PET: paired-end tag.

## Authors' contributions

WKS, GL, MJF and YR conceived the project and wrote the paper. GL and HX designed the algorithm and performed the research. GL, HX, FHM, SV, YBM, HSO, CT and PNA implemented the software. GL, FHM, MJF, YBM and SV tested the pipeline. All authors read and approved the final manuscripts.
